# Landscape and meteorological determinants of malaria vectors’ presence and abundance in the rural health district of Korhogo, Côte d’Ivoire, 2016–2018, and comparison with the less anthropized area of Diébougou, Burkina Faso

**DOI:** 10.1371/journal.pone.0312132

**Published:** 2024-10-21

**Authors:** Paul Taconet, Barnabas Zogo, Ludovic P. Ahoua Alou, Alphonsine Amanan Koffi, Roch Kounbobr Dabiré, Cedric Pennetier, Nicolas Moiroux

**Affiliations:** 1 MIVEGEC, CNRS, IRD, Université de Montpellier, Montpellier, France; 2 Institut Pierre Richet (IPR), Bouaké, Côte d’Ivoire; 3 Institut de Recherche en Sciences de la Santé (IRSS), Bobo-Dioulasso, Burkina Faso; Clinton Health Access Initiative, UNITED STATES OF AMERICA

## Abstract

**Background:**

Understanding how weather and landscape shape the fine-scale distribution and diversity of malaria vectors is crucial for efficient and locally tailored vector control. This study examines the meteorological and landscape determinants of (i) the spatiotemporal distribution (presence and abundance) of the major malaria vectors in the rural region of Korhogo (northern Côte d’Ivoire) and (ii) the differences in vector probability of presence, abundance, and diversity observed between that area and another rural West African region located 300 km away in Diébougou, Burkina Faso.

**Methods:**

We monitored *Anopheles* human-biting activity in 28 villages of the Korhogo health district for 18 months (2016 to 2018), and extracted fine-scale environmental variables (meteorological and landscape) from high-resolution satellite imagery. We used a state-of-the-art statistical modeling framework to associate these data and identify environmental determinants of the presence and abundance of malaria vectors in the area. We then compared the results of this analysis with those of a similar, previously published study conducted in the Diébougou area.

**Results:**

The spatiotemporal distribution of malaria vectors in the Korhogo area was highly heterogeneous and appeared to be strongly determined and constrained by meteorological conditions. Rice paddies, temporary sites filled by rainfall, rivers and riparian forests appeared to be the larval habitats of *Anopheles* mosquitoes. As in Diébougou, meteorological conditions (temperatures, rainfall) appeared to significantly affect all developmental stages of the mosquitoes. Additionally, ligneous savannas were associated with lower abundance of malaria vectors. *Anopheles* species diversity was lower in Korhogo compared to Diébougou, while biting rates were much higher. Our results suggest that these differences may be due to the more anthropized nature of the Korhogo region in comparison to Diébougou (less forested areas, more agricultural land), supporting the hypothesis of higher malaria vector densities and lower mosquito diversity in more anthropized landscapes in rural West Africa.

**Conclusion:**

This study offers valuable insights into the landscape and meteorological determinants of the spatiotemporal distribution of malaria vectors in the Korhogo region and, more broadly, in rural west-Africa. The results emphasize the adverse effects of the ongoing landscape anthropization process in the sub-region, including deforestation and agricultural development, on malaria vector control.

## Introduction

Malaria remains a major public health burden globally, with over 240 million cases in 2022 (1). Despite a major decrease in malaria cases in the last two decades, the progress has stalled since 2015 [1], even re-increasing in certain areas (e.g. + 10% between 2015 and 2022 in the West African sub-region [1]). Involved in such worrying trends are, among others, the widespread of resistance to insecticides used in public health and agriculture among malaria vectors (*Anopheles* mosquitoes), population growth, and environmental changes [2–6]. To reinvigorate progress, shifting from a global approach of prevention and curation where interventions are deployed regardless of the context, to a local approach where interventions are tailored to the local settings, is a key feature encouraged by the whole malaria community [5]. In particular, for vector control (VC), it is crucial to acquire a good knowledge of entomological situation at operational scale so as to better target the places and times of interventions using complementary tools to those widely used, e.g the Long-Lasting Insecticidal Nets (LLIN). Towards this aim, it is important to understand how the environment shapes the presence, abundance and diversity of the vectors at a local scale in the present, and how environmental alterations, such as climate or Land Use Land Cover (LULC) changes, may impact them in the future [4, 7, 8].

Among the environmental determinants of malaria transmission, weather and landscape play a critical role. Because they impact the bio-ecology of mosquitoes, these environmental features shape the diversity, presence, abundance, and spatiotemporal distribution of malaria vectors, and in-fine the risk of transmission of the disease [4, 8–10]. In this context, the identification of meteorological and landscape determinants of spatiotemporal heterogeneity in malaria vector abundance within a given spatial and temporal framework is an increasingly common research topic, supported in particular by the proliferation of high-resolution environmental satellite data [11–15]. These studies are useful for understanding the local bionomics of malaria vectors, predicting and mapping the spatiotemporal distribution of the anopheles mosquitoes in the area, and deploying locally tailored vector control tools. On a different note, to assess the potential impact of climate or LULC changes on the diversity or abundance of mosquitoes, it is common to compare such indicators between areas that have different environmental conditions [16–18]. In this study, we propose to use both approaches to better understand the landscape and meteorological determinants, and the impact of their middle and long-term change, in the presence, abundance and diversity of *Anopheles* mosquitoes in rural west Africa.

In a previous study [19], we investigated the environmental factors affecting the presence and abundance of the main malaria vectors at the scale of a west-African health district, in the Diébougou area, located southwestern Burkina Faso (BF). We used data on the spatiotemporal distribution and abundance of *Anopheles* mosquitoes collected in this area between 2016 and 2018 as part of a research project, together with landscape and meteorological data extracted from high-resolution Earth observation data, into a state-of-the-art statistical modeling framework. As part of the same project, *Anopheles* collections were carried out simultaneously 300 km away, in the Korhogo area, northern Côte d’Ivoire (incidence rate in 2022: 288 cases per thousand, mortality rate: 93 per 100 thousand [1]), using similar protocols. The goal of the present study was to replicate the modeling work in the Korhogo area with the aim to assess the meteorological and landscape conditions that affected the presence and abundance of the main malaria vectors in this area and to compare the results with what we previously found in Diébougou. From a methodological perspective, we discuss the added-value of consistent entomological surveillance data, used in conjunction with high resolution satellite data and powerful statistical modeling tools, to improve our understanding of the impact of climate and LULC changes on the diversity, abundance, and distribution of malaria vectors.

## Material and methods

### Study area

**Fig 1:**
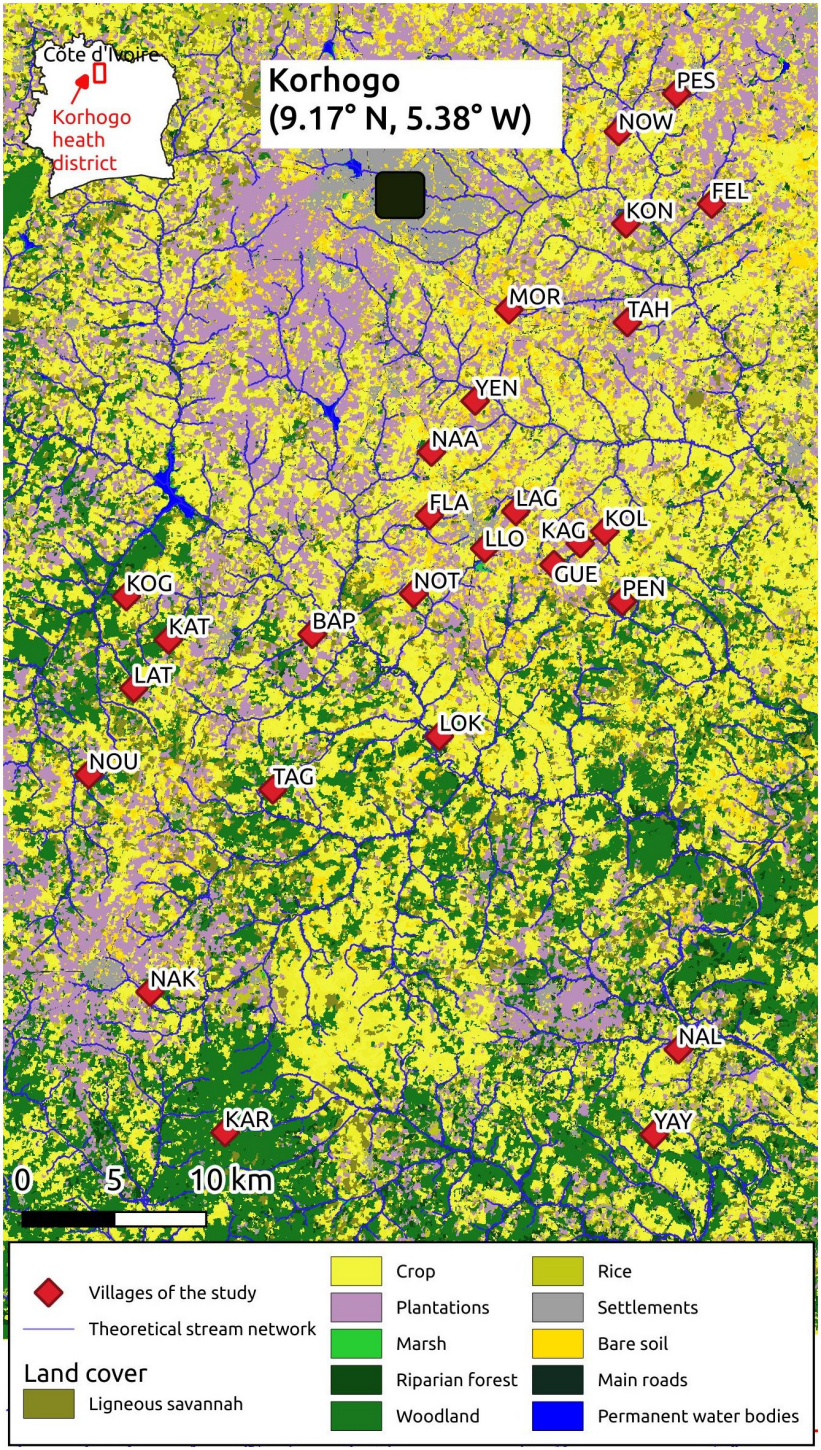
Map of the study area. The map includes the villages of the study, the land cover derived from very high spatial resolution satellite image acquired on 2017-10-11, and the theoretical stream network derived from a digital elevation model (see Methods section).

The Korhogo area is located in the north of Côte d’Ivoire (CI), in the Sudanian bioclimatic region [20]. The climate is characterized by a dry season from October to April (including a ’cold’ period from December to February and a ’hot’ period from March to April) and a rainy season from May to September. Average annual cumulated rainfall varies from 1 200 to 1 400 mm and daily temperatures vary from 21°C to 35°C. During the period covered by our study (from 2016-09-30 to 2018-03-24, i.e. 1.5 years), cumulated rainfall was 1 693 mm, with high variations between the dry and the rainy season (S1 Fig); average daily diurnal Land Surface Temperature (LST) was 35.1°C (SD = 3.9) and average daily nocturnal LST was 19.7°C (SD = 2.5). The landscape is mainly a mixture of agricultural lands (51% of the total surface of the study area) and natural vegetation (30%) (S1 Fig) [21]. Agricultural land is composed of croplands (including fallows) (24% of the total surface of the study area), cashew and mango plantations (18%), and rice paddies (9%). Natural vegetation is mainly composed of woodlands (17% of the total surface of the study area), savannas (9%) and riparian forests (4%). The region is dotted with villages of a few hundred people each, and has a high density of hydraulic small dams (see Fig 1) that allow for year-round agriculture. Regarding vector control, the primary VC tool is the LLIN, distributed universally by the government every 3–4 years since 2010 [22]. The last distribution before the beginning of the REACT project was in 2014. During the project, LLINs were distributed in the study villages in June 2017. As part of the REACT project, complementary VC tools were implemented in some of the villages in the middle of the project—namely indoor residual spraying of insecticide, intensive Information Education and Communication to the populations, and larval control.

### Entomological data

As part of the REACT project conducted concurrently in the rural health districts of Diébougou (BF) and Korhogo (CI), Anopheles human-biting activity was monitored [23]. A total of 28 villages covering an area of 70 by 40 km were chosen for the Korhogo area (Fig 1) based on the following criteria: each village had 200–500 residents, it was accessible during the wet season, and there was a minimum of 2 km between the villages. The names and geographic coordinates of the villages (both in the CI and BF area) are available at [23]. Eight rounds of mosquito collection were conducted in each village between October 2016 and March 2018. The periods of the surveys span some of the typical climatic conditions of this tropical area (2 surveys in the “dry-cold” season, 3 in the “dry-hot” season, 3 at each extremum of the rainy season; S1 Fig). During each survey, mosquitoes were collected one night, from 17:00 to 09:00, both indoors and outdoors, at four sites per village using the Human Landing Catch (HLC) sampling method. To conduct the HLC, community agreement were received before the beginning of the study, and written informed consent were obtained from all the mosquito collectors and supervisors. Mosquito collectors and supervisors were recruited from the beginning of the study (first HLC session: 2016-09-21) to its end (last HLC session: 2018-04-03). During the collections, all the mosquitoes were placed in individual hemolysis tubes plugged with cotton, stored in hourly bags. Malaria vectors were further identified using morphological keys [24]. Then, all individuals belonging to the Funestus Group and a sub-sample of the individuals belonging to the Gambiae Complex (due to the very large number of individuals collected) were identified to species using molecular analyses [25, 26]. The sub-sampling strategy was as following: for the first four survey, one individual of the Gambiae Complex randomly selected per hour per collection site (indoors/outdoors) in six randomly selected villages (over 28) was selected and proceeded for molecular identification. For the remaining four surveys, a random sub-sample representing 25% of the individuals belonging to the Gambiae Complex was proceeded for molecular identification. Collection design for this study has been extensively described in [27], and the data are available in the Global Biodiversity Information Facility (GBIF) [28].

### Landscape and meteorological data

Landscape variables were extracted from a very high spatial resolution (1.5-meter) LULC map of the study area containing 16 classes, that was produced *ad-hoc*. The raster map and the detailed methodology used to generate it are available at [21]. From this map, we merged under-represented and/or similar classes: “dense forest” and “open forest” were merged into a single class of non-riparian forests named “woodland”, and “cashew plantation” and “mango plantation” were merged into a single class named “plantation”. We calculated the percentage of landscape occupied by each land cover class in four spatial buffer areas around each collection site (250 m, 500 m, 1 km, and 2 km buffer radii).

We generated the theoretical stream network in the study area using a Digital Elevation Model [29] from which we computed two variables: the length of streams in each buffer zone and the shortest distance from each collection point to the stream network. We calculated the Clark and Evans aggregation index [30] (clustering of the households in each village) and the distance from each collection point to the edge of the village that are proxys of the attractiveness and penetrability of the villages for malaria vectors.

Meteorological variables (temperatures and rainfall) were extracted from satellite imagery. The Moderate Resolution Imaging Spectroradiometer (MODIS) Land Surface Temperature (LST) Terra and Aqua products were used to calculate the daily diurnal and nighttime temperatures [31, 32], and rainfall estimates were extracted from the Global Precipitation Measurement (GPM) Integrated Multi-satellitE Retrievals for GPM (IMERG) Final products [33]. These meteorological data were gathered up to 42 days (6 weeks) before each entomological survey, so as to cover largely the whole duration of the anopheles life cycle in the field [34]. They were then aggregated pixel-by-pixel on a weekly scale, averaged in a 2-km radius buffer zone around each HLC collection point, and finally cumulated (rainfall) or averaged (temperature) for all possible time lags between 0 and 6 weeks preceding the collection dates.

More details on the methods used to generate the landscape and meteorological variables can be found in [19].

### Statistical analyses

We used a hurdle-like methodology to model the malaria vectors’ biting rates: we modeled separately the probability of human-vector contact and the positive counts of human-vector contact—respectively called « presence » and « abundance » models in the rest of this article. In the presence models, the response variable was the presence/absence of vectors (binarized as 1/0) collected during 1792 nights of HLC (28 villages *8 entomological surveys * 4 collection points * 2 locations), while in the abundance models, the response variable was the number of bites per human on the positive catch sessions only—i.e. the sessions with at least one bite. In addition, we modeled the biting rates separately for each vector species, as they might exhibit different ecological preferences. Since two main vector species were found (see section Results), four response variables were hence built in total (presence and absence of both *An*. *gambiae s*.*s*. and *An*. *funestus*).

For each of the response variables, we used a two-stage statistical approach (a bivariate and a multivariate analysis, described below), each potentially providing complementary information on *Anopheles* bioecology.

#### Bivariate analysis

We calculated the Spearman correlation coefficient between the response variable and each environmental variable taken at the different buffer zones (for the landscape variables) and time lags (for the meteorological variables); with the aim of identifying the distances (around the capture point) and the periods (prior to capture), respectively, for/in which our environmental variables had the greatest effect on biting rates. For the meteorological variables, we generated Cross Correlation Maps (CCM) [35] to study the influence of environmental conditions during multiple time intervals (instead of single time points) prior to the collection event.

#### Multivariate analysis

Beginning with all the environmental variables created, we first selected a subset of variables to include in the multivariate model using the following variable selection algorithm: we excluded variables that were poorly correlated with the response variable (correlation coefficients < 0.1 or p-values > 0.2) (except for variables related to the presence of water which were all retained). Then, for each of the remaining meteorological (or landscape) variable, we retained the variable with the time lag interval (or buffer radius) showing the higher absolute correlation coefficient value. We further excluded collinear variables (i.e. Pearson correlation coefficient > 0.7) based on empirical knowledge. We included two adjustment variables in the models: the vector control tool(s) used and the place of collection (indoors or outdoors). These variables may influence the presence and abundance of the species but will not be discussed in this study, since we focus here on environmental determinants. Selected variables were used to train a multivariate Random Forest (RF) model [36] (binary classification and regression RF for the presence and abundance models, respectively) following the same method as previously described [19]. The predictive power of each model was assessed by spatial leave-one-village-out cross-validation, measuring the ability of the models to predict biting rates on out-of-sample, unseen nights of HLC. Precision—recall (PR) plots were generated for the presence models, and precision—recall area under the curve (PR-AUC) was calculated and compared to a baseline PR curve. Sensitivity and specificity were also calculated. For abundance models, visual evaluation through (i) distribution of mean absolute errors and (ii) observed versus predicted values for each out-of-sample village was preferred due to expected low performance metrics given the overdispersion of the response variables and the type of model used (i.e. non-parametric model) [37].

To interpret the strengths and shapes of associations learned by the RF models, we generated Variable Importance Plots (VIP) [37] to estimate the environmental variables that were the most influential in determining the behavior of the response variable and Partial Dependence Plots (PDP) [38] to estimate the functional relationship between each environmental variable and the response variable.

More details on the statistical framework used in this study can be found in [19].

### Comparison with the Diébougou area

As mentioned in the Introduction and in the Entomological data sections, the human biting activity of *Anopheles* mosquitoes was monitored as part of a research project (the REACT project) carried out simultaneously in the rural health districts of Korhogo (CI) and Diébougou (BF). Results from the Diébougou area, obtained from data and analyses following the same methods that those presented here, were published previously in [19].

With the aim of discussing similarities and differences between the results obtained in the Diébougou area of Burkina Faso [19] and those of this replication work in the Korhogo area of Côte d’Ivoire, we used key indicators to summarize the landscapes, the meteorological regimes, and their association with the spatiotemporal distribution of vectors. The selected indicators were the % area occupied by each land cover class (in the whole area and in the 2 km buffer around the collection points), cumulated rainfall, nocturnal (minimum) and diurnal (maximum) weekly temperature as well as *Anopheles* richness, diversity (Shannon’s index) and average measured biting rates. A set of results from the bivariate and multivariate analyses was extracted (correlation coefficients and time lag showing the higher correlation coefficient from the CCMs, most important variables from the VIP in the multivariate models).

### Ethics approval and consent to participate

Ethical clearance for the study was granted by the National ethics committee (No. 063/MSHP/CNER-kp) in Côte d’Ivoire and by the Institutional Ethics Committee of the Institut de Recherche en Sciences de la Santé (No. A06/2016/CEIRES) in Bukina Faso. We received community agreement before the beginning of the study, and we obtained written informed consent from all the mosquito collectors and supervisors. Yellow fever vaccines were administered to all the field staff. Collectors were treated free of charge when they were diagnosed with malaria during the study period according to WHO recommendations. They were also free to withdraw from the study at any time without any consequences.

## Results

### Specific composition and spatiotemporal distribution of *Anopheles* biting rates

A total of 1792 human-nights of collections was conducted in the Korhogo area (28 villages * 8 surveys * 4 points * 2 locations). A total of 57 716 anopheles were collected (representing over 90% of all mosquitoes collected), of which 56 267 (97.5%) and 714 (1.2%) belonged to the Gambiae Complex and the Funestus Group, respectively. Over the 3922 *An*. *gambiae s*.*l*. individuals (7% of the total) selected for molecular identification, 3726 (95%) were *An*. *gambiae s*.*s*. and 196 (5%) were *An*. *coluzzii*. Consequently, throughout the remainder of the manuscript, we will consider *An*. *gambiae s*.*l*. as *An*. *gambiae s*.*s*.

*An*. *gambiae s*.*s*. and *An*. *funestus* were present (i.e. at least one individual captured) respectively in 64% and 6% of the human-nights of collections. The distribution of positive human biting rates (i.e. human-nights with at least one bite) was highly left-skewed (for *An*. *gambiae s*.*s*.: median (med) = 18 bites/human/night, standard deviation (sd) = 65, maximum (max) = 505; for *An*. *funestus*: med = 2, sd = 12, max = 84). Additional details on *Anopheles* bionomics, *Plasmodium falciparum* sporozoite infection, and insecticide resistance mechanisms for this set of mosquitoes are provided in other publications [39, 40].

**Fig 2:**
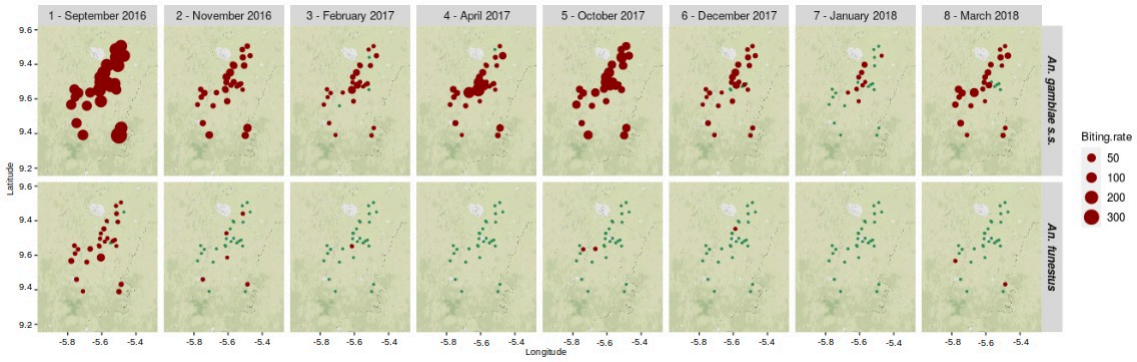
Map of the biting rates of the two main vector species for each village and entomological survey. Unit: average number of bites/human/ night. Blue dots indicate absence of bites in the village for the considered survey. Background layer: OpenStreetMaps.

Fig 2 shows the spatiotemporal distributions of the biting rates of the main *Anopheles* species. The map shows that *An*. *gambiae s*.*s*. was more abundant during or at the end of the rainy season (September, October) than in the dry season, when it was nevertheless present. Spatially, we note i) a certain level of heterogeneity in the distribution, and ii) that the species was present in almost all villages in all entomological surveys (except the 7th). The spatiotemporal distribution of *An*. *funestus* was very unbalanced: the overwhelming majority of individuals (93%) were collected during the first entomological survey, and almost half of the individuals (42%) were collected in a single village.

### Bivariate analysis

**Fig 3:**
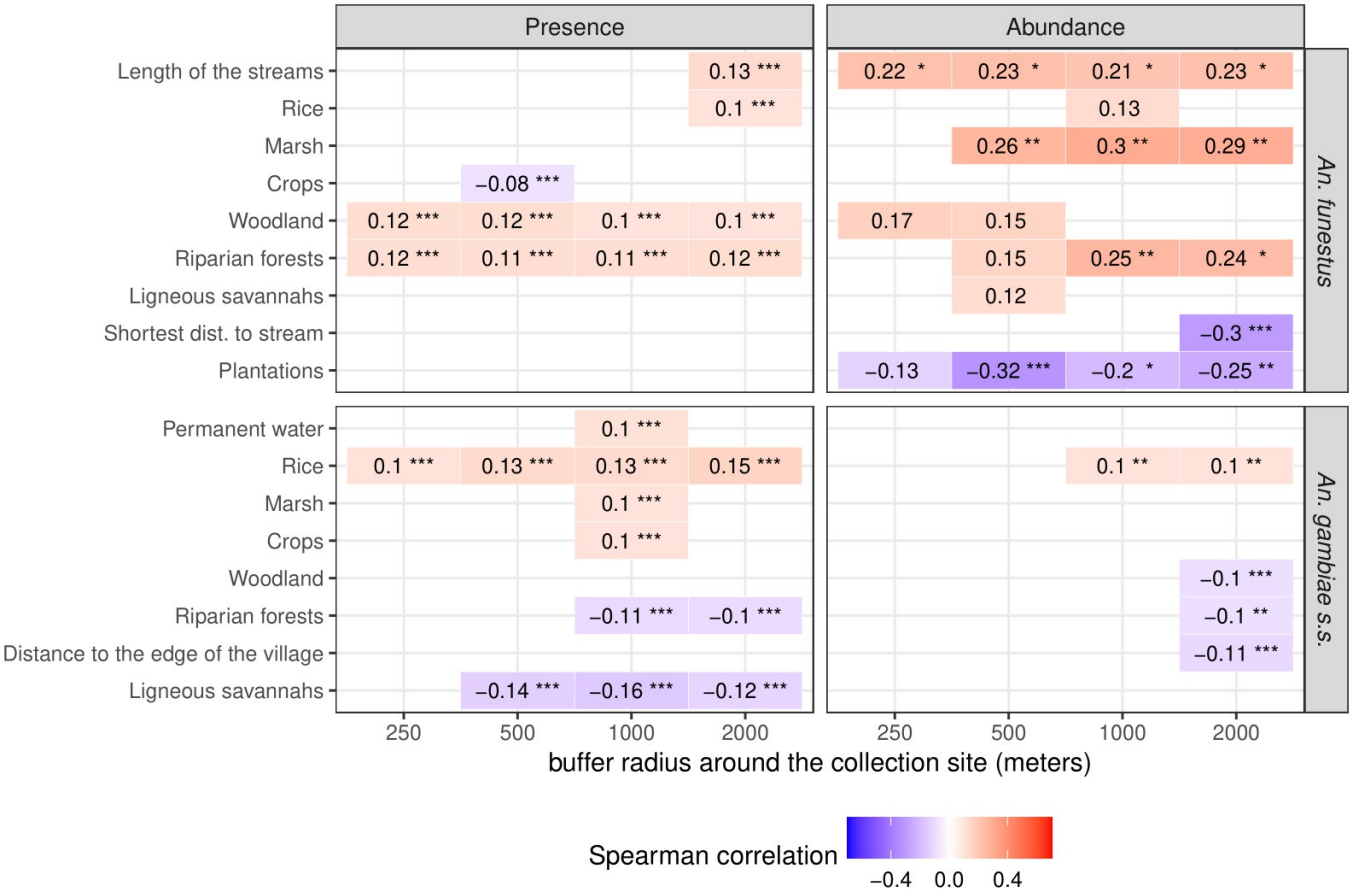
Plot showing the Spearman correlation between the vectors’ biting rates and the landscape variables. Landscape variables were extracted within four spatial buffer zones around the sampling locations (250 m radius, 500 m, 1 km, 2 km), and the Spearman correlation coefficient between these variables and biting rates was computed for each primary vector species. The analysis was divided into the presence/absence of bites (left) and the abundance of bites (i.e., positive counts only) (right). Landscape variables depict the percentage of surface occupied by each land cover class in each buffer zone. Only correlations with coefficient > 0.1 and p-values < 0.2 are displayed. Stars indicate the range of the p-value: *** p-value ∈ [0, 0.001[; ** p-value ∈ [0.001, 0.01[; * p-value ∈ [0.01, 0.05[; absence of stars: p-value ∈ [0.05, 0.2[.

Fig 3 shows the landscape variables that were significantly correlated (Spearman correlation coefficient (cc) > 0.1 and *p*-value < 0.2) with the presence or abundance of the studied vector species. The presence and abundance of *An*. *funestus* were correlated with more landscape variables than that of *An*. *gambiae s*.*s*., and the highest correlation coefficients with the landscape variables were found for *An*. *funestus*.

The presence of *An*. *funestus* was positively correlated with the length of the rivers and with the % of surface area occupied by rice paddies areas, in the 2-km radius buffer zone. It was also correlated with the % of surface occupied by riparian forests and woodland (i.e. non-riparian forest areas) in all buffer zones. The abundance of the species was positively correlated with the length of the streams and the percentage (%) of surface occupied by rice paddies areas, marshlands, riparian forests, and (non-riparian) forest areas, in various buffer zone sizes according to land cover class. The abundance of *An*. *funestus* was negatively correlated with the % of surface occupied by croplands in the 2-km radius buffer zone, and with the distance to the nearest stream (i.e. abundance was higher when the collection point was closer to the hydrographic network).

The presence of *An*. *gambiae s*.*s*. was positively correlated with the % of surface occupied by permanent water, marshlands, and crop areas in the 1-km radius buffer zone. The presence and abundance of the species were also correlated with the % of surface occupied by rice paddies areas, in all the buffer zones for presence and in the 1-km and 2-km radius buffer zones for abundance. The presence and abundance of *An*. *gambiae s*.*s*. were negatively correlated with the % of surface occupied by riparian forests, in the 1-km and 2-km radius buffer zones for presence and in the 2-km radius buffer zone for abundance. The abundance of the species was negatively correlated with the % of surface occupied by forested areas in the 2-km radius buffer zone, and with the distance to the edge of the village (i.e. abundance was higher in dwellings located near the edge of the village than in those close to the center of the village). The presence of *An*. *gambiae s*.*s*. was negatively correlated with the % of surface occupied by ligneous savannas in all buffer zones with radius > 250 m.

**Fig 4:**
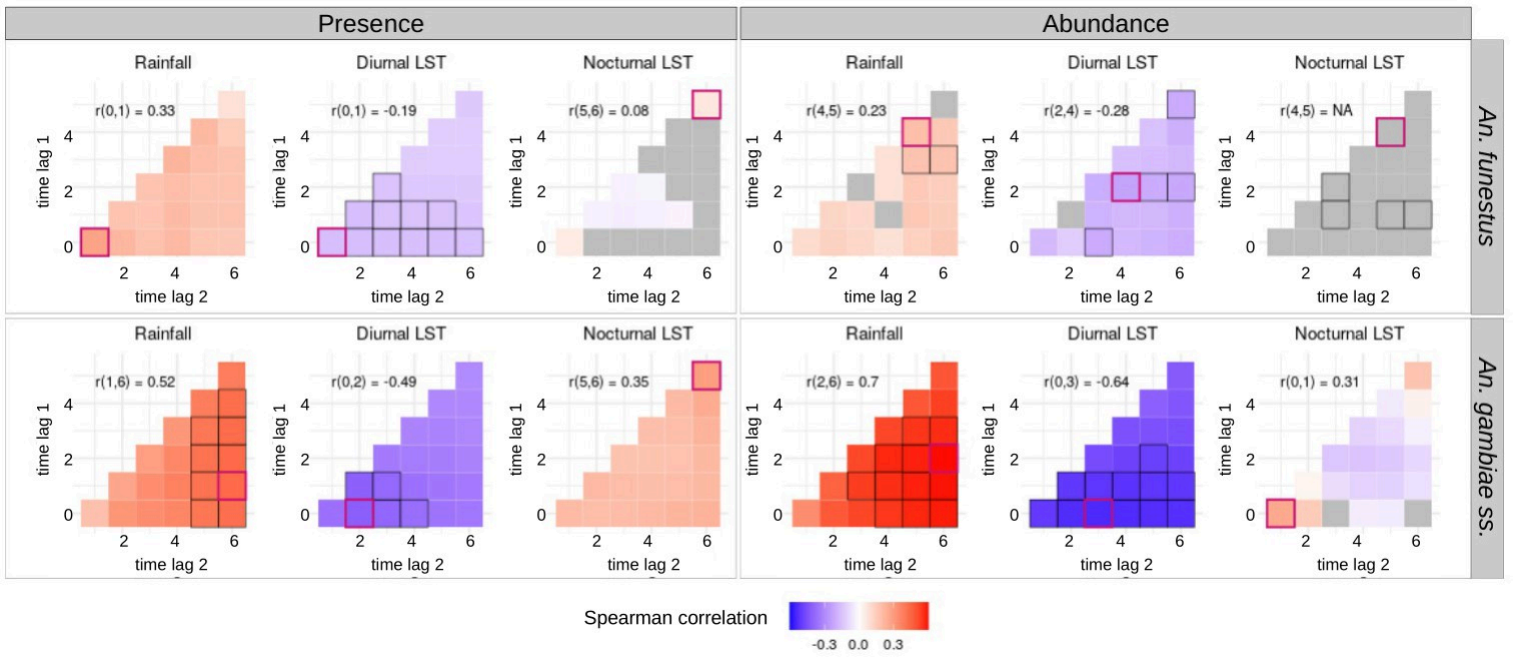
Cross-correlation maps (CCMs) showing the Spearman correlation between the vectors’ biting rates and the meteorological variables. To construct these CCMs, meteorological variables were gathered on a weekly scale up to 6 weeks before the collection dates, and the Spearman correlation coefficient between these variables and biting rates was computed for each main vector species. The analysis was separated into the presence/absence of bites (left) and the abundance of bites (i.e. positive counts only) (right). In each CCM, time lags are expressed in week(s) preceding the date of collection. The red-bordered square highlights the time lag interval exhibiting the highest correlation coefficient (absolute value) with the specific meteorological variable. The associated time lag interval and correlation coefficient are presented in the top-left corner of the CCMs. Black-bordered squares denote correlations close to the highest observed correlation (i.e., less than a 10% difference). Gray-filled squares indicate non-significant or low correlation (i.e., p-value > 0.2 or coefficient < 0.1).

Fig 4 shows the meteorological variables that were significantly correlated (spearman correlation coefficient (cc) > 0.1 and *p*-value < 0.2) with the presence and abundance of the vector species (in the form of cross-correlation maps).

The presence and abundance of *An*. *funestus* were positively correlated with cumulated rainfall preceding the date of collection, at almost all time lags. The presence and abundance of the species were negatively correlated with daytime temperatures, again at almost all time lags preceding collection. The correlations between the presence or abundance of *An*. *funestus* and the nocturnal temperatures preceding the date of collection were weak or non-significant.

The presence and abundance of *An*. *gambiae s*.*s*. were positively and strongly correlated with the cumulative rainfall preceding collection, at all time lags. The time interval showing the maximum correlation coefficient with cumulative rainfall was between 1 and 6 weeks before the date of collection for presence, and between 2 and 6 weeks before the date of collection for abundance. The presence of *An*. *gambiae s*.*s*. was also positively correlated with the nocturnal temperatures preceding the date of collection at all time lags, with the highest correlation coefficient observed for interval between 5 and 6 weeks before the date of collection. The presence and abundance of *An*. *gambiae s*.*s*. was negatively correlated with the diurnal temperatures preceding the date of collection, at all time lags. The maximum correlation coefficient between diurnal temperatures and the presence/abundance of the species was found between 0 and 2–3 weeks before the date of collection.

The correlation coefficients between the presence/abundance of species and the meteorological variables were higher for *An*. *gambiae s*.*s*. than for *An*. *funestus*.

### Multivariate analysis

**Fig 5:**
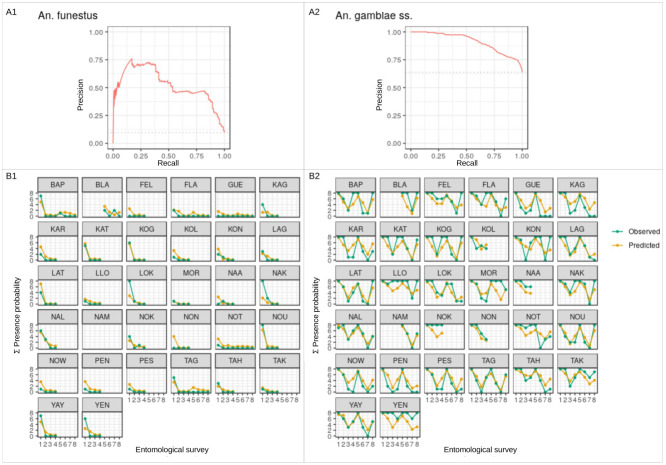
Model evaluation plots for the presence models. A1 and A2 depict precision-recall curves corresponding to the presence models of *An*. *funestus* and *An*. *gambiae s*.*s*. Precision-recall curves illustrate the precision and recall of the models across various probability thresholds for the ’presence’ class. Precision signifies the accuracy of presence identifications, while recall indicates the proportion of actual presence observations correctly identified. The dashed horizontal line represents the baseline (random or no-skill) classifier, and a precision-recall curve above this line indicates a classifier performing better than no-skill. The area between the precision-recall curve and the baseline line signifies the classifier’s effectiveness, with a larger area indicating superior performance. Plots B1 and B2 showcase observed versus predicted presence probabilities for each out-of-sample village. The y-axis reflects the sum across the eight sampling points per village per survey (four points per village at two positions, interior and exterior). Overall, A1 and A2 reveal that the models demonstrated strong predictive capabilities, as their precision-recall curves surpass the baseline. Additionally, B1 and B2 demonstrate that the models accurately predicted the spatiotemporal trends of presence/absence of mosquito bites, with lines of predicted presence probabilities closely aligning with those of observed probabilities.

**Fig 6:**
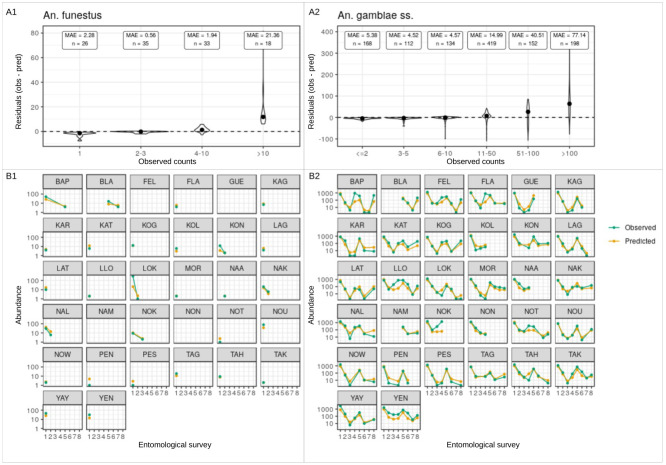
Model evaluation plots for the abundance models. A1 and A2 present violin plots illustrating the distribution of residuals in the abundance models for *An*. *funestus* and *An*. *gambiae s*.*s*., categorized by observed counts of mosquito bites. The median values are denoted by black dots. B1 and B2 display observed versus predicted numbers of bites per village per entomological survey. The y-axis reflects the total number of bites across the eight sampling points per village per survey (four points per village at two positions, interior and exterior), presented on a logarithmic scale. The absence of a dot indicates the absence of collected vectors. MAE (mean absolute error) is noted, and n denotes the number of observations. Overall, A1 and A2 reveal that the models effectively predicted small to medium observed counts of bites, as evidenced by low MAEs and small residuals, which constitute the majority of observations (high n). Larger counts (>50 bites) tended to be underestimated by the models. B1 and B2 affirm these findings and further demonstrate the accurate prediction of general trends in biting rates over time, with the lines representing predicted abundance closely tracking those of observed abundance.

The Precision-Recall area under the curve (PR-AUC) of the presence models were 0.52 (baseline = 0.09) and 0.91 (baseline = 0.64) for *An*. *funestus* and *An*. *gambiae s*.*s*. respectively. The specificity and sensitivity of the models at the optimal probability thresholds were respectively 53% and 98% for *An*. *funestus* and 88% and 61% for *An*. *gambiae s*.*s*. These results suggest good predictive power for the presence models. Both species’ spatiotemporal trends were accurately predicted by the abundance models, despite the fact that high counts were frequently understimated. Model evaluation plots are presented in Fig 5 (presence models) and Fig 6 (abundance models).

**Fig 7:**
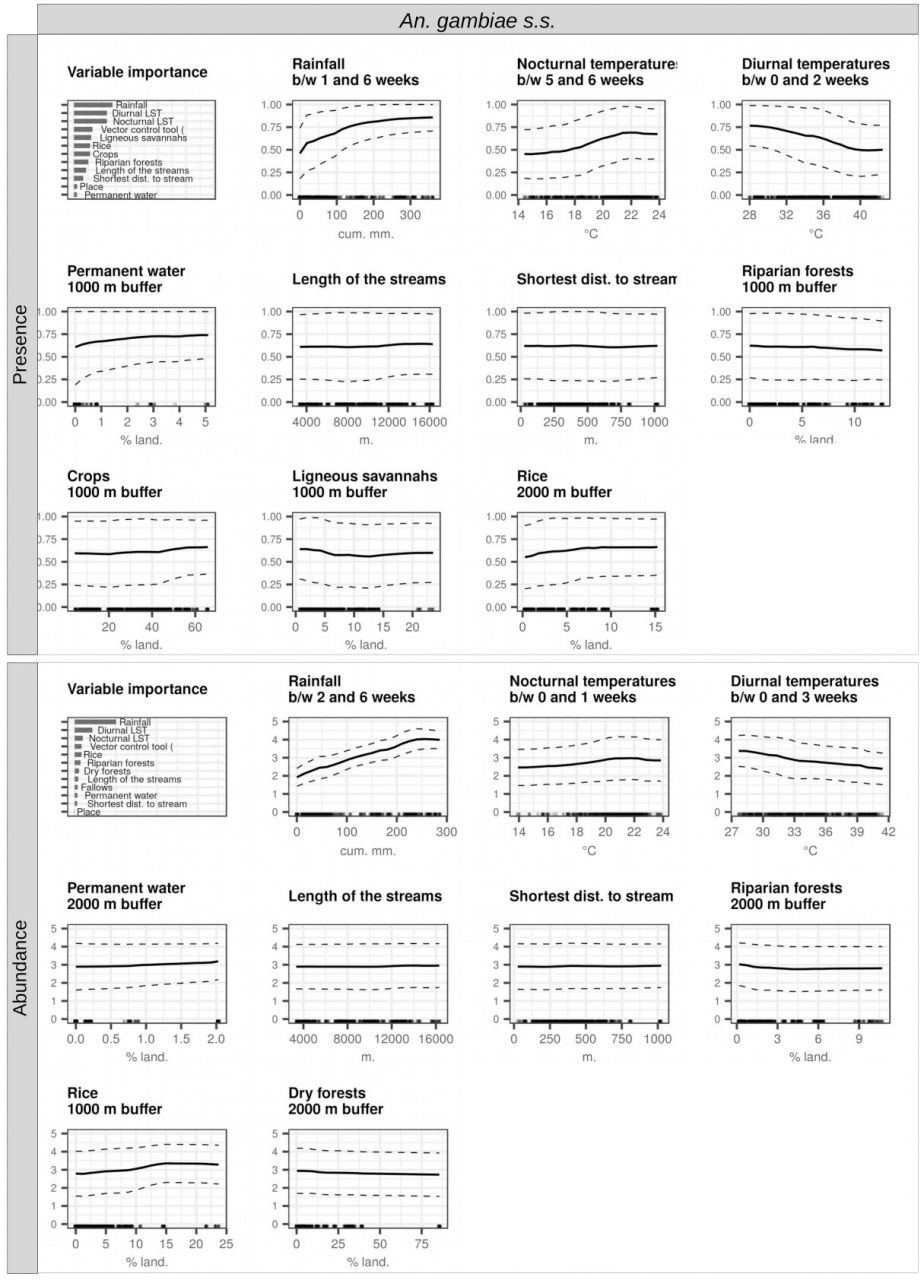
Interpretation plots of the random forest models for the presence and abundance of *An*. *gambiae s*.*s*. Biting rates were categorized into the presence/absence of bites and the abundance of bites (i.e., positive counts only), leading to the creation of two models: presence (top) and abundance (bottom). In each model, the top-left corner plot serves as the variable importance plot. The remaining plots consist of partial dependence plots (PDPs) for each variable incorporated into the models (one plot per variable). The y-axis in the PDPs signifies, in the presence models, the probability of at least one individual biting a human during a night, and in the abundance models, the log-transformed number of bites received by one human in one night, conditioned on their presence. The dashed lines within the plots represent the partial dependence function ± one standard deviation, providing an indication of variability estimates. The x-axis values cover the range available in the data for the respective variable, and the rugs above the x-axis depict the actual values present in the data for that variable. Noteworthy abbreviations include LST for land surface temperature and between for between.

**Fig 8:**
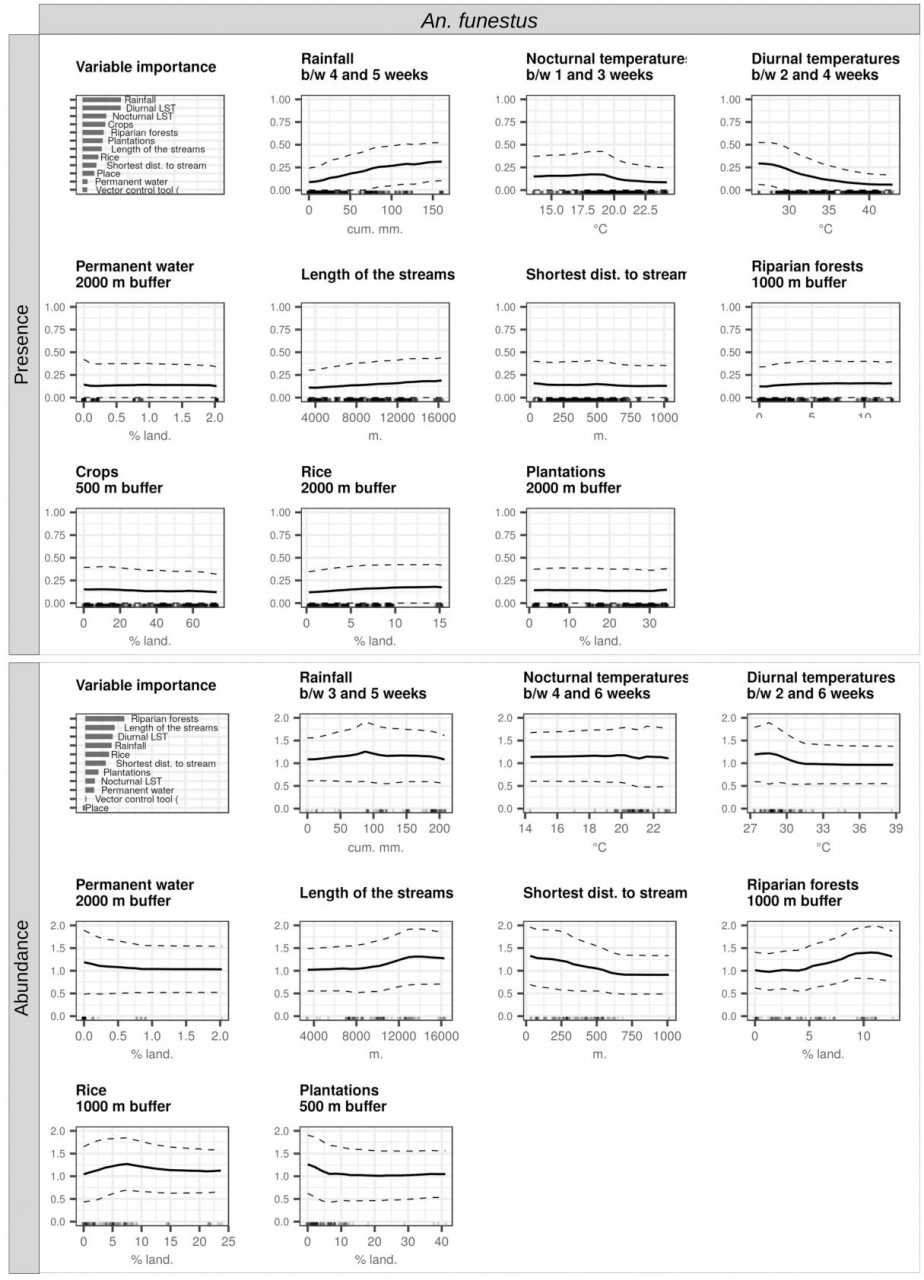
Interpretation plots of the random forest models for the presence and abundance of *An*. *funestus*. Biting rates were categorized into the presence/absence of bites and the abundance of bites (i.e., positive counts only), leading to the creation of two models: presence (top) and abundance (bottom). In each model, the top-left corner plot serves as the variable importance plot. The remaining plots consist of partial dependence plots (PDPs) for each variable incorporated into the models (one plot per variable). The y-axis in the PDPs signifies, in the presence models, the probability of at least one individual biting a human during a night, and in the abundance models, the log-transformed number of bites received by one human in one night, conditioned on their presence. The dashed lines within the plots represent the partial dependence function ± one standard deviation, providing an indication of variability estimates. The x-axis values cover the range available in the data for the respective variable, and the rugs above the x-axis depict the actual values present in the data for that variable. Noteworthy abbreviations include LST for land surface temperature and between for between.

Figs 7 and 8 show the model interpretation plots (variable importance plot and partial dependence plots) for *An*. *gambiae s*.*s*. and *An*. *funestus*, respectively.

The most important predictors of both the presence and abundance of *An*. *gambiae s*.*s*. were the three meteorological variables recorded during the weeks preceding collection (Fig 7): in order, cumulative rainfall between 1–2 and 6 weeks before collection (positive relationship), diurnal temperatures between 0 and 2–3 weeks before collection (negative relationship), and nocturnal temperatures (between 5 and 6 weeks and between 0 and 1 week before collection for the presence and abundance models, respectively) (positive relationship). It should be noted (i) that for the presence model, the three most important predictors were equally important, and (ii) that the importance of rainfall was particularly high in the abundance model, far outweighing the importance of all other variables.

The most important predictors of the presence of *An*. *funestus* were the three meteorological variable recorded during the weeks preceding collection (Fig 8): cumulative rainfall (positive relationship), diurnal temperatures (negative relationship), and nocturnal temperatures (negative relationship). The most important variables in the abundance model for this species were: the % of surface occupied by riparian forests (postive relationship), the total length of hydrographic stream in the 2-km radius buffer zone around the collection points (postive relationship), and diurnal temperatures (postive relationship).

### Comparison with the Diébougou area in Burkina Faso

Table 1 presents a set of selected indicators characterizing the landscape, meteorological regime, and entomological conditions in the Diébougou (BF) and the Korhogo (CI) area, as well as the impacts of the environmental (landscape + meteorological) conditions on the spatiotemporal distribution of vectors (i.e. main outputs of the bivariate and multivariate statistical analyses).

**Table 1:** Quantitative characterization of the landscape, meteorological, and entomological conditions in the Diébougou (BF) and the Korhogo (CI) area, and of the impacts of environmental conditions on the spatiotemporal distribution of vectors in each area.

	Diébougou (BF)	Korhogho (CI)
**Meteorological regime**		
Cumulated rainfall over the study period	1112 mm	1693 mm
Average diurnal LST (SD) over the study period	37.9°C (4.9)	35.1°C (3.9)
Average nocturnal LST (SD) over the study period	19.4°C (2.7)	19.7°C (2.5)
**Landscape**		
% natural vegetation	71%	30%
% agricultural lands	26%	51%
% rice paddies	1%	9%
% plantations	0%	17%
% savannah	52%	9%
% woodland	3%	18%
**Entomological conditions**		
*Anopheles* species richness	10	7
*Anopheles* species diversity (Shannon index)	1.23	0.33
Average human biting rate (bites/human/night)	1.98	32.21
**Associations between presence / abundance of vectors and environmental variables: time lag with higher cc in bivariate analysis)**		
Rainfall with presence of *An*. *gambiae s*.*s*.	2 to 6 weeks (0.33)	1 to 6 weeks (0.52)
Rainfall with abundance of *An*. *gambiae s*.*s*.	2 to 6 weeks (0.34)	2 to 6 weeks (0.7)
Diurnal LST with presence of *An*. *gambiae s*.*s*	0 to 2 weeks (-0.37)	0 to 2 weeks (-0.49)
Diurnal LST with abundance of *An*. *gambiae s*.*s*	0 to 2 weeks (-0.26)	0 to 3 weeks (-0.64)
Nocturnal LST with presence of *An*. *gambiae s*.*s*.	4 to 6 weeks (0.39)	5 to 6 weeks (0.35)
Nocturnal LST with abundance of *An*. *gambiae*	3 to 6 weeks (0.34)	0 to 1 weeks (0.31)
Rainfall with presence of *An*. *funestus*	1 to 3 weeks (-0.18)	0 to 1 week (0.33)
Rainfall with abundance of *An*. *funestus*	2 to 3 weeks (-0.14)	4 to 5 weeks (0.23)
Diurnal LST with abundance of *An*. *funestus*	3 to 6 weeks (-0.22)	0 to 1 weeks (-0.19)
Diurnal LST with abundance of *An*. *funestus*	4 to 6 weeks (-0.24)	2 to 4 weeks (-0.28)
Nocturnal LST with presence of *An*. *funestus*	0 to 3 weeks (-0.18)	5 to 6 weeks (0.08)
‍Nocturnal LST with abundance of *An*. *funestus*	1 to 3 weeks (-0.19)	ns
**Associations between presence / abundance of vectors and environmental variables: most important variables in multivariate analysis (ordered by importance)**		
Presence model for *An*. *gambiae s*.*s*.	Nocturnal LST Diurnal LST Rainfall	Rainfall Diurnal LST Nocturnal LST
Abundance model for *An*. *gambiae s*.*s*.	Rainfall Diurnal LST Marshlands	Rainfall Diurnal LST Nocturnal LST
Presence model for *An*. *funestus*	Grassland Marshlands Ligneous Savannah	Rainfall Diurnal LST Nocturnal LST
Abundance model for *An*. *funestus*	Marshlands Grassland Ligneous Savannah	Riparian forest Hydrographic stream Diurnal LST

These indicators were extracted from the landscape, meteorological and entomological data described in the Methods section, and the bivariate and multivariate statistical models described in the Methods section as well. LST = land surface temperature, cc = correlation coefficient, pp = percentage point. Time lags are displayed only if p-values<0.2 and cc>0.1, either ns is displayed.

The Diébougou area is located in the Sudanian bioclimatic region, as Korhogo. The seasonality is characterized by the same dynamics (i.e. dry-cold, dry-hot, and rainy season). During the study period (from 2016-09-30 to 2018-03-24), cumulated rainfall was 1112 mm (-34% compared to Korhogo) (S3 Fig); average diurnal Land Surface Temperature (LST) was 37.9°C (sd = 4.9) (+2.8°C compared to Korhogo) and average nocturnal LST was 19.4°C (sd = 2.7) (-0.3°C compared to Korhogo). The landscape in the Diébougou study area was, as for Korhogo, mainly a mixture of natural vegetation and agricultural lands [41], with some notable differences (S4 Fig). Agricultural land accounted for 26% of the total surface area of the Diébougou study area, proportionally half that of Korhogo. Rice paddies represented only 1% of the total surface area in Diébougou (against 9% in Korhogo), and there was no tree plantations in the Diébougou study area (17% of the study area in Korhogo). Conversely, the natural vegetation accounted for 71% of the total surface, proportionally more than twice that of Korhogo. It was composed of savannas (52% of the total surface), grasslands (7%), marshlands (5%), riparian forests (4%), and woodlands (3%). Contrary to Korhogo, the Diébougou area had very few hydraulic dams.

Vector control strategies implemented in the Diébougou area were similar to those of Korhogo. The primary vector control tool is the LLIN. The last distribution before the beginning of the REACT project was in 2016 [42]. As part of the REACT project, complementary VC tools were implemented as well in some villages.

In the Diébougou area, three main *Anopeheles* species were collected: *An*. *gambiae s*.*s*., *An*. *coluzzii*, *An*. *funestus* [27]. The specific composition was more balanced than in the Korhogo area (20% *An*. *gambiae s*.*s*., 44% *An*. *coluzzii*, 24% *An*. *funestus*). The *Anopheles* species richness was higher than in Korhogo (10, against 7 in Korhogo) as well as the diversity (Shannon index = 1.23, against 0.33 in Korhogo). The average human biting rate was much lower (1.98 bites/human/night in Diébougou vs. 32.21 bites/human/night in Korhogo). The distribution of the vector species in space and time was highly heterogeneous, as in Korhogo (S5 Fig and Fig 1 in [19]). However in Diébougou, *An*. *funestus* was present in a higher ratio of villages and entomological surveys. The seasonal dynamics of *An*. *gambiae s*.*s*. were similar to those of the Korhogo area but, unlike the Korhogo area, the species was completely absent from most of the villages during the dry season.

In the bivariate analysis, the absolute values of the correlation coefficients between the presence/abundance of *Anopheles* species and the landscape variables were overall lower in the Korhogo area than in the Diébougou (BF) area (see Fig 3 in [19]). Conversely, the absolute values of the correlation coefficients between the presence/abundance of the species and the meteorological variables were generally higher in the Korhogo area than in the Diébougou area, particularly for *An*. *gambiae s*.*s*. (see Fig 4 in [19]). Notably, the CCMs of *An*. *gambiae s*.*s*. in the Korhogo and Diébougou areas were, one by one, very similar: while the absolute values of the correlation coefficients were generally slightly higher in the Korhogo area, the time lags with the highest correlation correlation coefficients were almost identical for 5 of the 6 CCMs.

The presence and abundance models had high predictive power in the Diébougou area, as for the Korhogo area (see Additional files 4 and 5 in [19]). Overall, the most important predictors of the presence and abundance of *An*. *gambiae s*.*s*. in the Diébougou area were, as for Korhogo, the meteorological variable recorded during the weeks preceding collection (see Fig 7 in [19]). The secondary predictors were also close to those of Korhogo (e.g. marshlands, riparian forests, ligneous savannas)–although rice paddies were not present. In contrast to Korhogo, the most important predictors of the presence of *An*. *funestus* in Diébougou were landscape variables (marshlands, grasslands, savannas) (see Fig 6 in [19]).

## Discussion

The overarching aim of this study was to investigate (i) the landscape and meteorological determinants of the spatiotemporal distribution of the major malaria vectors in the rural Korhogo region (Côte d’Ivoire), and (ii) the differences with another rural West African region, the Diébougou (Burkina Faso) area.

### Landscape and meteorological determinants of the presence and abundance of malaria vectors in the Korhogo area

In the Korhogo rural area in Côte d’Ivoire, rainfall was the most important predictor of both the presence and abundance of *An*. *gambiae s*.*s*; directly followed by land surface temperature-related variables. The cross-correlations maps (CCM) of *An*. *gambiae s*.*s*. showed that the presence and abundance of the species were significantly correlated with the three meteorological variables at all time lags preceding collection. Similar observations were made in the Diébougou area in Burkina Faso [19]. These findings suggest that in the Korhogo area, as in Diébougou and also more widely in Africa [43], i) *An*. *gambiae s*.*s*. was dependent on temporary breeding sites filled by rainfall and ii) its life traits (development and survival at both larval and adult stages) were strongly impacted by temperatures, as more detailed in [19]. Moreover, some CCMs showed a maximum correlation with meteorological variables recorded at time periods anterior to the mean lifetime of collected mosquitoes (i.e. more than 3 weeks before collection). This suggests, as discussed in [19], that vector abundance and presence may have been influenced by the effect of weather on life traits of the parent generations (further impacting the collected generation through mechanical effects on population dynamics), or by preparing different biotic and abiotic conditions that affected the survival and development of the observed generation.

Our studies have revealed important similarities in the shapes of the CCMs of *An*. *gambiae s*.*s*. in the two study areas. Indeed, the *An gambiae s*.*s*. populations in Korhogo and Diébougou shared common time lag for the effect of weather on their dynamic. This finding suggests that population dynamics of *An*. *gambiae s*.*s*. in relation to the meteorological factors are highly comparable in these two areas, and by extrapolation, possibly in the entire sub-region where meteorological regimen are similar.

The % of surface occupied by rice paddies was the second and first most important landscape variable in the models of presence and abundance of *An*. *gambiae s*.*s*., respectively, suggesting that rice paddies were probably important breeding sites for *An*. *gambiae s*.*s* larvae, and enabled their year-round presence. This hypothesis was actually confirmed by a field study carried out in the Korhogo area by the REACT project team with the aim of characterizing the larval habitats of *Anopheles spp* [44]. In this latter study, the authors identified that rice paddies were the most frequent breeding sites for *An*. *gambiae s*.*s*., both during the rainy and dry seasons. Several studies, in the Korhogo areas [45] and elsewhere in West Africa [46, 47] had previously found that extension of irrigated rice cultivation was correlated to the density of the main malaria vectors.

The % of surface occupied by ligneous savannas around the villages was the most important landscape variable in the abundance model of *An*. *gambiae s*.*s*., with a negative correlation. This finding agrees to observations made in southern Côte d’Ivoire [10], in Benin [48] and in the Diébougou area [19]. It supports the hypothesis that the degree of openness of the surrounding landscape affects the biting rates of *An*. *gambiae s*.*s*. in the villages. Closed landscapes (in comparison to open landscape) may reduce the dispersal capacity of *Anopheles* mosquitoes [49], resulting in longer gonotrophic cycle duration, in turn leading to decreased biting frequencies [50]. Another plausible hypothesis is that closed landscapes may be less favorable to larval breeding as a consequence of lower sunlight exposure [44, 51], lower temperature [50] and possibly higher negative biotic interactions (competition, predation) [52, 53].

In the Korhogo area, unlike in Diébougou, the most important variables in the model of presence of *An*. *funestus* were all meteorological (in particular rainfall and diurnal temperature). Thus, contrary to the observations made in the Diébougou area, landscape was not the main driver of the spatiotemporal presence of *An*. *funestus* in the Korhogo area. On the other hand, when *An*. *funestus* was present, landscape strongly impacted its abundance (two of the three most important variables in the species abundance model were landscape-related), as in Diébougou. In particular, the species seemed particularly dependent on rivers and riparian forests. These landscape features therefore seemed to constitute preferential breeding sites for *An*. *funestus* in the Korhogo area, confirming the literature [43, 54].

The multivariate models correctly predicted the presence and abundance of the two species, as in Diébougou. This indicates that the main determinants of the presence and abundance of both species were identified and incorporated into the models.

### Differences in *Anopheles* mosquitoes diversity, presence and abundance between the Diébougou and Korhogo areas: The effect of meteorological conditions and landscape anthropization?

The Korhogo and Diébougou areas are « contextually » close: they are both rural areas in west Africa, located in the same bioclimatic region, distant only 300 km as the crow flies, and implementing similar VC strategies. Despite these similarities, we found notable differences in richness, diversity, and abundance of the malaria vectors. *Anopheles* species richness and, even more so, diversity were lower in Korhogo than in Diébougou. In addition, the average proportion of positive sessions (i.e. sessions with at least one mosquito collected) and the overall biting rates were much higher in the Korhogo area than in Diébougou. The present study, combined with that of Diébougou [19], offers insights into the potential reasons for these variations.

Our studies have demonstrated the significant impact of weather and landscape conditions on the presence and abundance of *Anopheles* mosquitoes in our study areas. Differences in both meteorological regimen and LULC in the two areas could explain these contrasts. The higher rainfall in Korhogo than in Diébougou may result in more numerous or persistent temporary breeding sites, the preferred habitat for larvae of several *Anopheles* species [43]. In terms of landscape, permanent larval habitats (rice paddies, dams irrigating them) were more abundant in Korhogo than in Diébougou. Furthermore, these habitats enabled the year-round presence of *Anopheles* larvae [44]. In contrast, ’closed’ natural environments (especially ligneous savannas)—which our models suggest reduce vector biting rates—were less common in Korhogo than in Diébougou. Overall, as a result of these differences, adult vectors and biting rates are likely to be higher.

The observed differences in *Anopheles* species richness and diversity could also be explained by the differences in landscape composition between the two areas, particularly the variation in natural vegetation cover. The Diébougou area, which is over 70% covered by natural vegetation (against 30% in the Korhogo area), has the potential to host a greater variety of mosquito species due to their species-specific preferences for different types of habitats, blood and sugar sources [43], that are more common in natural environments and particularly in woodland areas [6]. Overall, as stressed out by [55], woodland has the highest levels of species diversity on land, and almost all taxonomic groups are more likely to occur as woodland cover increases. These disparities in landscape composition (surface of rice paddies, number of dams, surface of savanna, etc.), which could explain the observed differences in malaria vectors presence, abundance and diversity, also indicate a higher level of anthropization of the land in the Korhogo area than in the Diébougou area. Our results hence support the hypothesis of higher vectors’ densities and lower mosquito diversity in more anthropized landscapes, as suggested by a recent meta-analysis of the link between landscape anthropization and mosquito diversity and abundance at a global scale [6]. A recent study conducted in western Burkina Faso has shown similar trends (i.e. fewer species in environments with high human impact, such as urban areas and rice fields, than in environments with lower human impact such as forested areas) [56].

The second, maybe less documented in the literature, is the removal of natural ‘closed’ natural environments (like ligneous savannas and forests), which seemed to act as protective barriers in both areas, especially when located closely around the villages. As stated previously, another recent study carried out in Côte d’Ivoire found a similar result [10]. A third process, whose effect could unfortunately not directly be assessed here, is the creation of artificial dams for agriculture. In this study, we could not directly assess their impact on mosquito presence and abundance because few dams were located in the considered 2-km radius buffer area around the collection points. However, these artificial infrastructures have already been identified as important breeding sites for anopheles mosquitoes in Africa in general [57–59] and in the Korhogo [44] and Diébougou [19, 60] areas in particular.

Anthropization could consist of replacing elements of the landscape that reduce biting rates (e.g. natural ligneous savannas) with elements that favour them (e.g. rice fields), thus cumulating the entomological impact. In practice, such processes are happening in West Africa: the sub-region has lost—and is still losing—large extents of its natural land cover classes, replaced by a heavily human-influenced landscape dominated by agriculture [20]. For example, Côte d’Ivoire lost 60% (-22,000 km^2^) of its forest in 38 years (1975 to 2013), while increasing agricultural lands, in the same period, by 84% (+31,600 km^2^) (20). Burkina Faso shows similar trends, with a 39% loss of savannas and 160% increase in rain-fed agricultural land over the same period [20].

It is important to note that while landscape anthropization may pose significant threats to the control of malaria vectors in rural West Africa, its impact on malaria transmission is less straightforward—as it might come with positive side-effects. For example, higher vector abundance associated with the development of irrigated crops may be associated with changes in biting patterns or life history of the vectors, or may be offset by the socioeconomic and public health improvements associated with agriculture [46]. In the Korhogo area, a study from 2003 showed that the extent of flooded surfaces associated to the extension of irrigated rice cultivation was strongly correlated to the density of the main malaria vector, but that there was no clear correlation between malaria transmission and these flooded surfaces, most probably due to the influence of intra-specific competition on the lifespan of the mosquito population [45]. Comparison of the malaria transmission indicators between our study areas or holistic statistical modeling of malaria incidence (including data related to the demographical, socio-economical, entomological, environmental, human behavioral, etc. contexts) could enable to better assess the interplay between vector abundance and malaria transmission risk.

### Limitations and directions for future work

The identification of the determinants of the presence and abundance of malaria vectors in the study area has several limitations, which have already been addressed and discussed in our previous work [19]. These limitations include: absence of ground-truth evaluation of the theoretical stream network dataset, absence of variables representing fine-scale potential important drivers of mosquito presence, abundance or biting rates (e.g. alternative sources of blood meal, domestic breeding sites, market gardening, water quality, etc.), absence of any study of interactions between variables, absence of confirmation of the cause-effect relations (i.e. bio-ecological processes) underlying the statistical correlations founds.

Our work paves the way for the development of operational tools to support the fight against malaria transmission in the Korhogo area. As detailed in our previous study [19], the knowledge and models generated in this study could support (i) conceptualization of tailored vector control intervention plans and tools, (ii) decisions regarding the places and times where recurrent (long-term) and (iii) occasional (short-term) interventions should be deployed. Although Côte d’Ivoire has begun implementing stratification of vector control at the district level in 2021 [61, 62], the heterogeneity in the spatial distribution of the malaria vectors in the Korhogo health district (but also in other districts [10, 63]) suggests that even more spatially stratified targeting of interventions, i.e. at the village level, would likely be beneficial. The VC operational tools mentioned above could be developed for the Korhogo and the Diébougou areas, but this study shows that they may also be applicable to much larger areas. Indeed, we identified several similarities in the predictive models from both areas (e.g. cross-correlation maps, relative importance of predictors, shape of relationships) that opens up interesting prospects for the generalizability of these models. Concretely, we could envisage, using the whole entomological dataset, to train a predictive model that could be used to predict the probability of presence and the abundance of *Anopheles* at the village level in rural areas beyond our two study areas (and further develop the related decision-making tools such as maps of predicted biting rates or EWS). The scalability of the models could actually be tested by attempting to predict the presence and abundance of *Anopheles* in the Diebougou area using the models trained in the Korhogo area, and vice versa. The exact spatial and temporal areas of applicability of such models remains to be determined.

Lastly, our work is an example of how harmonized entomological surveillance data, used in conjunction with high-resolution satellite data and powerful statistical modeling tools, can improve our understanding of the potential impact of climate and LULC changes on malaria vector density and, by extension, the role of environmental change in the stalling of malaria reduction progress that has been observed for almost a decade. Although fine-scale landscape and meteorological data covering the African continent are increasingly abundant and accessible, such research is limited by lack of consistent data on mosquitoes distribution in Sub-saharan Africa [64]. It should be remembered that the WHO now recognizes vector surveillance as a key feature of vector and malaria control [65] There is hence an urgent need for the implementation of mosquito surveillance systems that collect consistent, long-term, small-spatial-scale entomological data, and the development of an associated centralized, Findable, Accessible, Interoperable, and Reusable (FAIR) database. Recent technological developments in electronics, artificial intelligence, computer science and telecommunications show great potential for building surveillance systems with such features, for example by developing smart and connected mosquito traps that can autonomously count and identify mosquitoes and transmit the data wirelessly [66].

## Supporting information

S1 Fig Summary of the meteorological and landscape conditions in the Korhogo area during the mosquito collection period. A) Average meteorological conditions in a 2 km radius buffer zone around the collection points (weekly aggregation): Vertical red lines indicate the dates of the entomological surveys. Ribbons indicate the mean ± one standard deviation (i.e. spatial variability) considering all the sampling points for the date. Sources: for temperature: MODIS Land Surface Temperature (https://doi.org/10.5067/MODIS/MOD11A1.006), for rainfall: Global Precipitation Measurement (https://doi.org/10.5067/GPM/IMERGDF/DAY/06). B) Landscape conditions: Percentage of surface occupied by each land cover class i) in the whole study area (green bars) and ii) in a 2-km radius buffer areas around the collection points (orange bars). In the latter, error bars indicate the mean ± one standard deviation (i.e. spatial variability) considering all the sampling points. Source: https://doi.org/10.23708/MTF4S8.
(TIF)

S2 Fig Contextual map of the study areas (Korhogo in Côte d’Ivoire and Diébougou in Burkina Faso) and locations of the villages where entomological collections were performed between 2016 and 2018. (TIF)

S3 Fig Comparison of the meteorological conditions in the areas of Korhogo and Diébougou during the mosquito collection period. Average meteorological conditions in a 2 km radius buffer zone around the collection points (weekly aggregation) for the Korhogo and Diébougou areas. Vertical red lines indicate the dates of the entomological surveys (Korhogo area: orange lines, Diébougou area: grey lines). Sources: for temperature: MODIS Land Surface Temperature (https://doi.org/10.5067/MODIS/MOD11A1.006), for rainfall: Global Precipitation Measurement (https://doi.org/10.5067/GPM/IMERGDF/DAY/06).
(TIF)

S4 Fig Comparison of the landscape conditions in the areas of Korhogo and Diébougou during the mosquito collection period. A) Percentage of surface occupied by each land cover class in the whole study areas, for Korhogo area (orange bars) the Diébougou area (grey bar) B) Percentage of surface occupied by each land cover class in a 2-km radius buffer areas around the collection points in the Korhogo area (left plot) and in the Diébougou area (right plot). Sources: for Korhogo: https://doi.org/10.23708/MTF4S8, for Diébougou: https://doi.org/10.23708/ARSJNB.
(TIF)

S5 Fig Plots of the spatial and temporal distribution of the main malaria vectors species observed in the areas of Korhogo and Diébougou. (TIF)
