# Supplementary figures and images for: Landscape and meteorological determinants of malaria vectors’ presence and abundance in the rural health district of Korhogo, Côte d’Ivoire, 2016–2018, and comparison with the less anthropized area of Diébougou, Burkina Faso
Source: PLoS One. 2024 Oct 21;19(10):e0312132. doi: 10.1371/journal.pone.0312132 (PMC11493267; doi:10.1371/journal.pone.0312132)

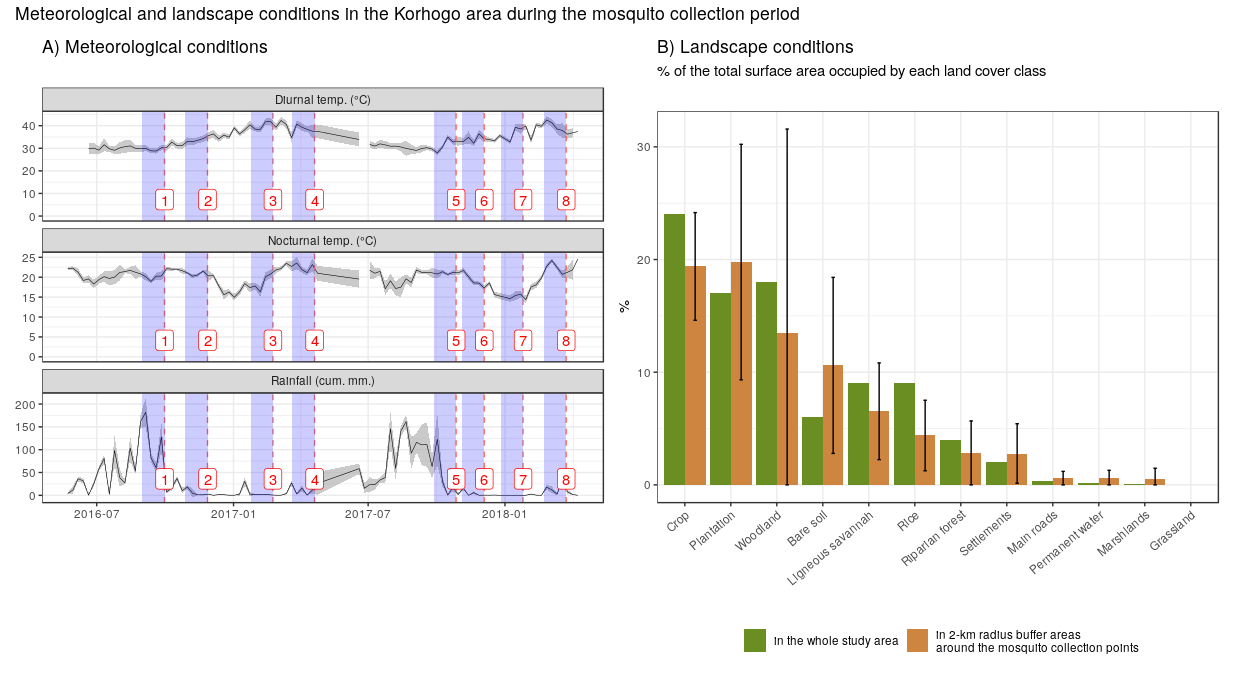

Supplement: S1 Fig — A) Average meteorological conditions in a 2 km radius buffer zone around the collection points (weekly aggregation): Vertical red lines indicate the dates of the entomological surveys. Ribbons indicate the mean ± one standard deviation (i.e. spatial variability) considering all the sampling points for the date. Sources: for temperature: MODIS Land Surface Temperature (https://doi.org/10.5067/MODIS/MOD11A1.006), for rainfall: Global Precipitation Measurement (https://doi.org/10.5067/GPM/IMERGDF/DAY/06). B) Landscape conditions: Percentage of surface occupied by each land cover class i) in the whole study area (green bars) and ii) in a 2-km radius buffer areas around the collection points (orange bars). In the latter, error bars indicate the mean ± one standard deviation (i.e. spatial variability) considering all the sampling points. Source: https://doi.org/10.23708/MTF4S8. (TIF) [file pone.0312132.s001.tif]

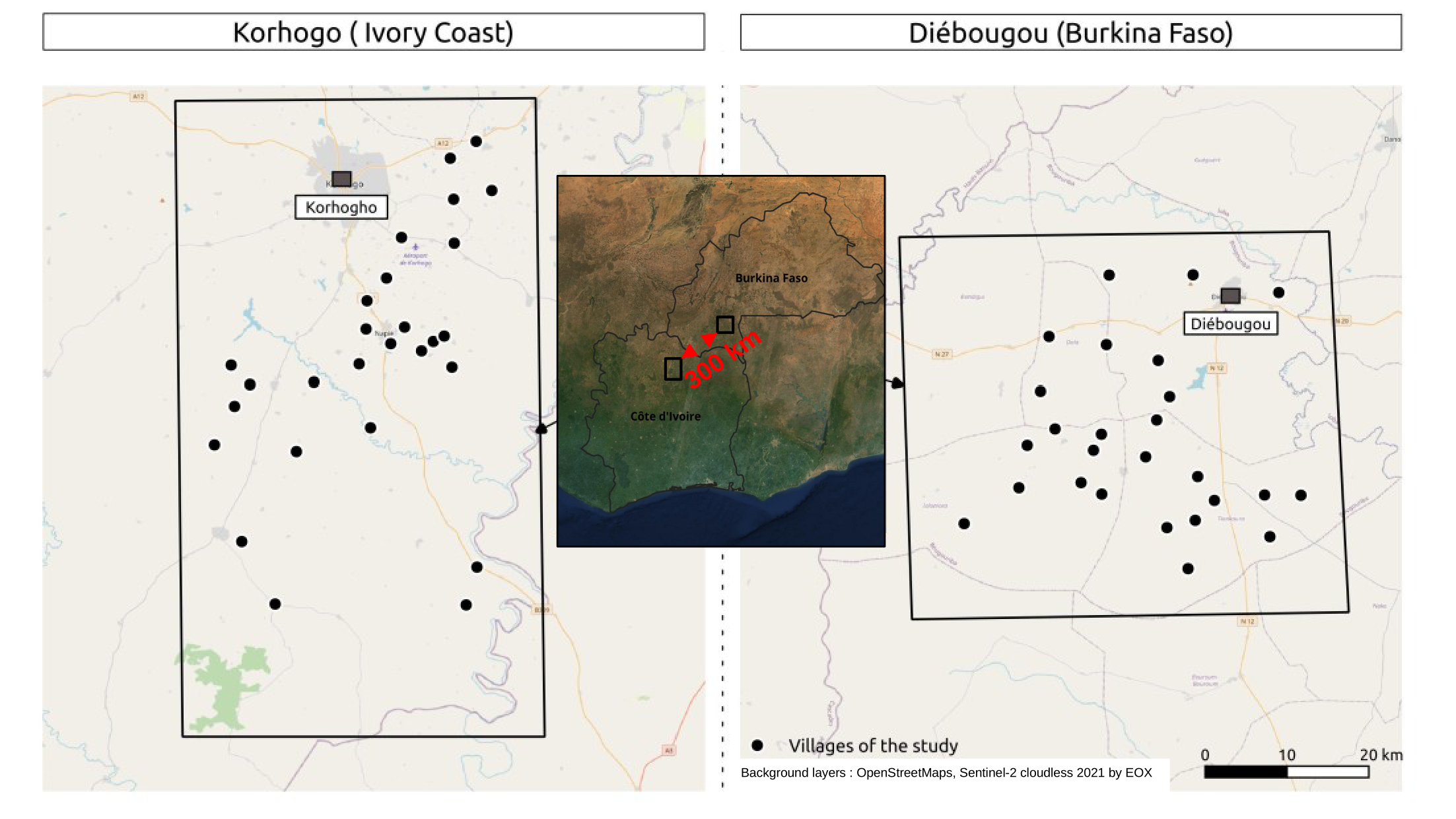

Supplement: S2 Fig — (TIF) [file pone.0312132.s002.tif]

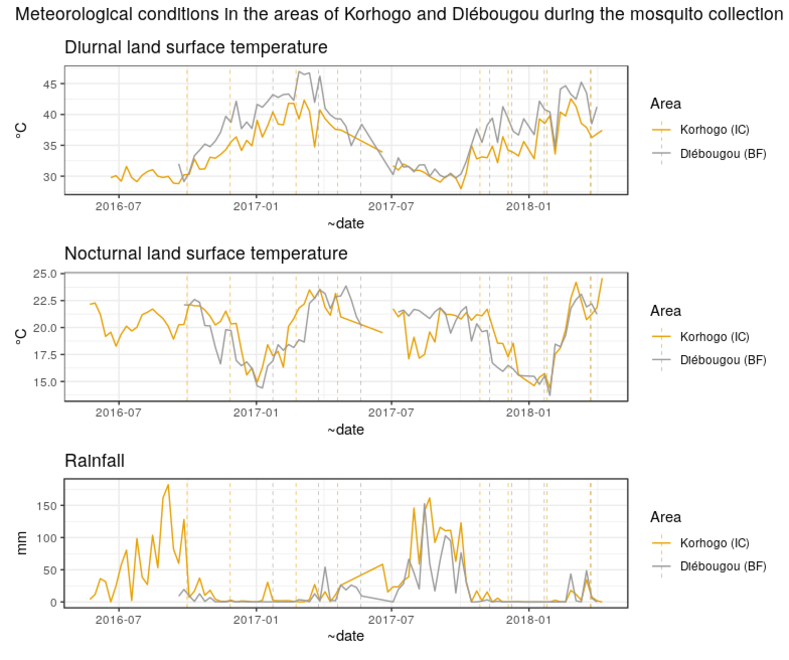

Supplement: S3 Fig — Average meteorological conditions in a 2 km radius buffer zone around the collection points (weekly aggregation) for the Korhogo and Diébougou areas. Vertical red lines indicate the dates of the entomological surveys (Korhogo area: orange lines, Diébougou area: grey lines). Sources: for temperature: MODIS Land Surface Temperature (https://doi.org/10.5067/MODIS/MOD11A1.006), for rainfall: Global Precipitation Measurement (https://doi.org/10.5067/GPM/IMERGDF/DAY/06). (TIF) [file pone.0312132.s003.tif]

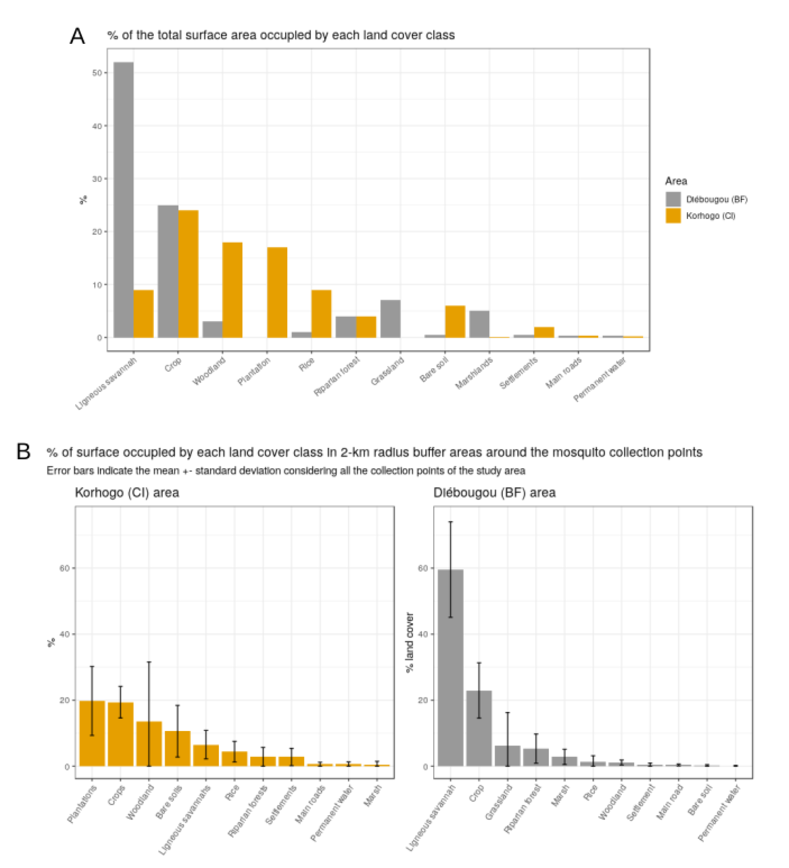

Supplement: S4 Fig — A) Percentage of surface occupied by each land cover class in the whole study areas, for Korhogo area (orange bars) the Diébougou area (grey bar) B) Percentage of surface occupied by each land cover class in a 2-km radius buffer areas around the collection points in the Korhogo area (left plot) and in the Diébougou area (right plot). Sources: for Korhogo: https://doi.org/10.23708/MTF4S8, for Diébougou: https://doi.org/10.23708/ARSJNB. (TIF) [file pone.0312132.s004.tif]

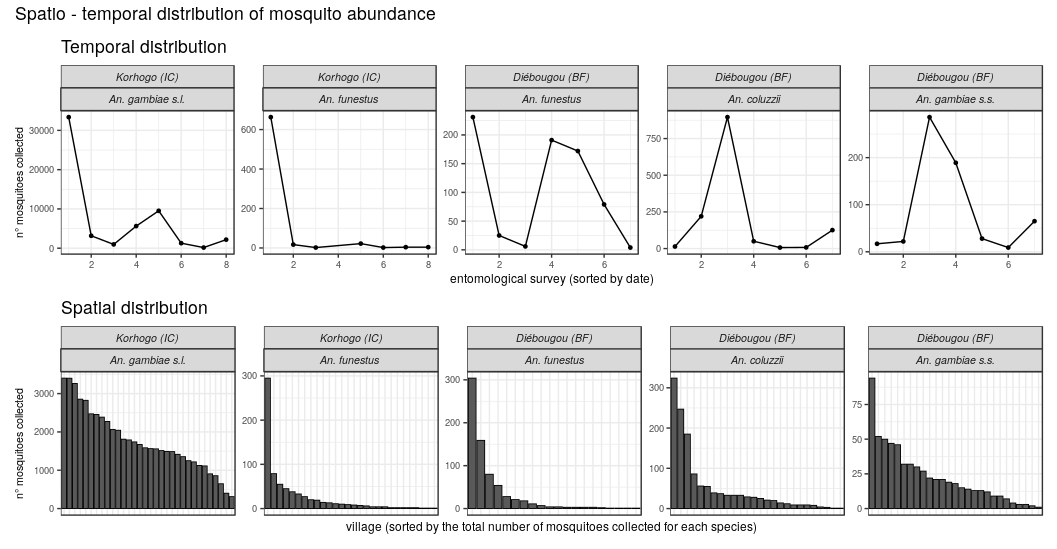

Supplement: S5 Fig — (TIF) [file pone.0312132.s005.tif]
