# Supplementary material for: Landscape and meteorological determinants of malaria vectors’ presence and abundance in the rural health district of Korhogo, Côte d’Ivoire, 2016–2018, and comparison with the less anthropized area of Diébougou, Burkina Faso
Source: PLoS One. 2024 Oct 21;19(10):e0312132. doi: 10.1371/journal.pone.0312132 (PMC11493267; doi:10.1371/journal.pone.0312132)
Supplement: Attachment — Submitted filename: PONE-D-24-07519_review.docx [file pone.0312132.s006.docx]

The presented study by Taconet *et al* examines the meteorological and landscape determinants of the spatiotemporal distribution of the major malaria vectors in the rural region of Korhogo and the differences in vector probability of presence, abundance, and diversity observed between that area and another rural West African.

I am impressed by the wealth of entomological data the authors have collected to carry out their study. I enjoyed reading the manuscript as the writing style is overall accessible and concise.

I’d recommend this manuscript for publication after the revision suggested below have been implemented:

**Abstract**

It is not advisable to use the personal pronouns ‘I’ and ‘we’ in a scientific document. It has been used throughout the document; the document should be reviewed and corrected.

**Introduction**

Include in the introduction section the current state of malaria in Côte d'Ivoire (incidence and mortality).

**Results**

I understand that the focus of this study is on malaria vectors. However, i suppose that, you collected extremely many *Culex* and *Aedes* mosquitoes. Their potential role in disease transmission, habitat competition is very important. I suggest that this be taken into account in the results.

Figures 2, 4, 5 and 6: Figures legend and axis are not clear.
