# Supplementary material for: Landscape and meteorological determinants of malaria vectors’ presence and abundance in the rural health district of Korhogo, Côte d’Ivoire, 2016–2018, and comparison with the less anthropized area of Diébougou, Burkina Faso
Source: PLoS One. 2024 Oct 21;19(10):e0312132. doi: 10.1371/journal.pone.0312132 (PMC11493267; doi:10.1371/journal.pone.0312132)
Supplement: Attachment — Submitted filename: response_to_reviewers.docx [file pone.0312132.s007.docx]

Dear Editor,

Thank you for taking the time to assess our manuscript “Landscape and meteorological determinants of malaria vectors’ presence and abundance in the rural health district of Korhogo, Côte d’Ivoire, 2016- 2018, and comparison with the less anthropized area of Diébougou, Burkina Faso”. We would also like to thank the two reviewers for their time, their positive assessment of the work, and their thoughtful feedbacks. The reviewer’s comments have helped us to further improve and sharpen the manuscript.

Below we provide the point-by-point responses to the reviewer’s comments. The cited references are provided at the end of the responses. **Please note that the line numbers refer to the lines in the revised manuscript with track changes.**

Sincerely,

Authors

**Reviewer #1:**

The paper is on the comparative study of environmental factors that determine the presence and abundance of malaria vectors in 2 areas with different degrees of anthropic activities.

The work was well carried out, and the data analyzed adequately with models. However there are certain aspects of “theoretical” data generated eg length of river, surface temperature etc which would benefit if some ground truthing or actual recording with data logger were done to confirm the data.

**1/ There is inherent risk in doing HLC, perhaps future work should not use HLC. Research in Malaysia now uses mosquito traps only.**

Thank you for this comment. We fully agree with you. In the subsequent project (REACT 2) that our team carried out in the same areas, we actually used BG mosquito traps to collect entomological data.

**2/ Another point is the mosquitoes were not tested if they contain malaria parasites, and whether those caught indoors are more likely to have parasites. Perhaps some published papers would have this information. This information would be useful to be included.**

In our project, quantitative PCR (qPCR) was performed on a sub-sample of the collected *Anopheles* spp*.* mosquitoes to screen them for *Plasmodium falciparum* sporozoite infection. The results (including the sporozoite infection rates per species and surveys, and the sporozoite infection rates in malaria vector populations according to location (indoors and outdoors)) are presented in Zogo et al., 2019a (see Tables 3 and 4 in that publication).

Here, we wanted to focus on the environmental determinants of the overall presence and abundance of malaria vectors in the study area. For this reason, we did not dwell into the details of biting behaviour and sporozoite infection.

However, we agree that readers of our manuscript might be interested in having these information. We hence added the reference to the aforementioned publication in the revised version of the manuscript, in the Results section (l. 302).

**3/ *144 … mosquitoes were collected one night, from 17:00 to 09:00, both indoors and outdoors, at four sites*
Were there any differences between the catch (species, abundance and time of catch) between indoors and outdoors?**

Yes, there actually were some differences among the catch (species, abundance and time of catch) between indoors and outdoors. The overall exophagy rate (% of bites received outdoor) was 56 % for *An. gambiae s.l.* and 69 % for *An. funestus*. The exophagy rate of *An. gambiae s.l.* varied little, both amongst the entomological surveys and the villages. The exophagy rate of *An. funestus* was more heterogeneously distributed in time and space. The early biting rate (i.e. % of bites received before 50% of the LLIN users were declared to be under their bednet at night) was 3% for *An. gambiae s.l*. and 13% for *An. funestus*. The early biting rate was overall stable among the surveys and villages for *An. gambiae s.l.* and was more heterogeneously distributed for *An. funestus*. The late biting rate (i.e. % of bites received after 50% of the LLIN users were declared to be out of their bednet in the morning) was lower than the early biting rate: 1% for both *An. gambiae s.l.* and *An. funestus* and was overall stable among the surveys and villages.

Part of these information are extracted from Taconet et al., 2024 (see e.g. Figure 2.A. and Table 2 in that publication). Zogo et al., 2019a provides complementary information on the overall biting patterns of malaria vector populations from these data.

**4/ *145 ... per village using the Human Landing Catch (HLC) sampling method*
Was only one catcher stationed per site?, was there any analysis of variation of catch between catchers? Between indoors and outdoors?**

There were two catchers per collection site (one indoor and one outdoor). The distance between indoor and outdoor collection points in one site was at least 10 m to minimize competition between mosquito collectors. As stated in the Method section (l. 159), collection design for this study has been extensively described in a data paper (Taconet et al., 2023), and the full dataset was published in the Global Biodiversity Information Facility (GBIF) (Soma et al. 2023). This is why we did not extended too much on the data collection method in the manuscript. Similarly, in the manuscript, we did not dwell on biting behavior (exophagy, early or late biting) as the main purpose here is the study of the environmental determinants of the overall presence and abundance of vectors. However, as stated previously, the overall bionomics of the malaria vectors (including exophagy/endophagy), and their potential determinants, were extensively studied elsewhere (Zogo et al., 2019a ; Taconet et al., 2024).

**5/ *153 for the first four survey, one individual of the Gambiae Complex randomly selected per hour per collection site (indoors/outdoors) in six randomly selected villages (over 28) was selected and ..*
Presumably the mosquitoes were kept separately for each hour? Maybe this could be explained in the text.**

Thank you for pointing out that we were not fully clear here. In fact, as explained data paper describing the mosquito data collection design (Taconet et al., 2023), mosquitoes collected were placed in individual hemolysis tubes plugged with cotton, stored in hourly bags. We have added this information in the revised version of the manuscript (l. 150).

**6/ *175 We generated the theoretical stream network in the study area using a Digital Elevation Model (29).*
Some ground truthing would be useful here.**

Thank you for this relevant comment. We agree with you that ground truthing of the stream network would be important, to get an evaluation of the generated theoretical stream network dataset (as was done for the land cover map). Unfortunately, we could not perform such ground truthing. To raise this important point in the revised version of the manuscript, we have added this lack of ground truthing as a limitation of the study in the Discussion section (l. 704).

**7/ *Multivate analysis. We first selected a set of environmental variables to introduce in the*
State what were the variables selected for testing.**

Thank you for pointing-out that this sentence is quite confusing. What we meant here is that, beginning with all the environmental variables created (described in the section *Method -> Landscape and meteorological data*), we first selected a subset of variables to include in the multivariate model using a variable selection algorithm (that we describe after in the text).

To clarify the sentence, we rephrased it in the revised version of the manuscript (l. 225).

**8/ *178 Over the 922 An. gambiae s.l. individuals (7% of the total)*
I think it should be “Over the 3922 …..**

Thank you for pointing out this error. We have now revised the manuscript as suggested (l. 294).

**9/ *319 The presence of An. funestus was positively correlated with the length of the rivers.*
The river length was generated theoretically, and the correlation may be correct. However the quality of river water is more important for the life cycle of the mosquitoes. Unfortunately, the quality of the river water cannot be calculated by satellite images.**

Indeed, as rightly noted, the quality of the water (including that of the rivers) is very important for the life cycle of the mosquitoes. In the frame of the REACT project, a field study aiming at identifying the *Anopheles* spp. breeding habitats was specifically performed by our team (Zogo et al., 2019b) in the Korhogo area. Various water quality indices (turbidity, presence of solid waste, liquid waste, vegetation, fish, etc.) were measured in the frame of this study, and linked to the presence and abundance of anopheles larvae.

Here, we did not directly measure the quality of the water, and therefore we were not able to create related independent variables. To raise this important point in the revised version of the manuscript, we have added this lack of data on the water quality as a limitation of the study in the Discussion section (l. 707).

**10/ *455 temperatures between 0 and 2-3 weeks before collection (negative relationship), and nocturnal temperatures (between 5 and 6 weeks and between 0 and 1 week before collection for the presence and abundance models, respectively) (positive relationship).*
I am not sure if explanation was given.**

Thank you for this remark. The biological hypotheses underlying the associations between presence/abundance of vectors and diurnal (maximal) / nocturnal (minimal) temperatures were extensively described in the ‘mirror’ study (Taconet et al., 2021), in Burkina Faso, that is cited all throughout the manuscript. In that manuscript, we wrote :

*“For both species [*An. gambiae s.s. and An. coluzzii*], diurnal temperature had the strongest effect during the 2 weeks preceding the dates of collection. This indicates that increasing diurnal temperatures probably reduced adult survival and larval development rates of the sampled generation of mosquitoes, leading to lower observed abundance. Indeed, high temperatures are known to inhibit development of anopheline larvae and to reduce adult survival. Regarding nocturnal temperatures, the time period with the strongest effect on the presence and abundance of both An. gambiae s.s. and An. coluzzii was between 3 and 6 weeks before collection. This indicates that nocturnal (i.e. minimal) temperatures had their strongest impact by either affecting previous generations (low temperatures are known to reduce adult survival and inhibit larval development) or modifying habitats (with a delay) for the collected generation (for instance, low temperatures may inhibit the development of algae, whose biomass has been found to be associated with larval densities).”*

In the present study, we provided a summarized explanation for these associations in the Discussion section : *“[*An. Gambiae s.s.*] life traits (development and survival at both larval and adult stages) were strongly impacted by weather conditions”.* However, we fully agree with you that these explanations are not clear enough. To clarify this point, in the revised version of the manuscript, we have (l. 572) :

- replaced “*weather conditions” with “temperatures” ;*

- referred to the Taconet et al., 2021 publication.

**11/ *569 The % of surface occupied by rice paddies*
The % should be spelled out.**

Thank you for pointing out this inconsistency. We have now revised the manuscript as suggested (l.341).

**Reviewer #2:**

The presented study by Taconet et al examines the meteorological and landscape determinants of the spatiotemporal distribution of the major malaria vectors in the rural region of Korhogo and the differences in vector probability of presence, abundance, and diversity observed between that area and another rural West African.
I am impressed by the wealth of entomological data the authors have collected to carry out their study. I enjoyed reading the manuscript as the writing style is overall accessible and concise.
I’d recommend this manuscript for publication after the revision suggested below have been implemented:

**1/ Abstract - It is not advisable to use the personal pronouns ‘I’ and ‘we’ in a scientific document. It has been used throughout the document; the document should be reviewed and corrected.**

Thank you for your remark. The use of personal pronouns in scientific articles is an important debate : see e.g. the following interesting ressources that summarize the research in this field : Kedia G, 2023 ; Wordvice KH, 2023.

Altogether, these ressources suggest that there is no clear consensus on the use of personal pronouns in scientific articles, but nevertheless, that they are not banned – and may even be encouraged in some cases, e.g. : to describe the approach the authors choose to follow (“In this study, we explore…”, “First, we will discuss…”), to describe procedures (“We conducted the experiment…”), to present the findings (“We found that…”), to avoid anthropomorphism (“We observed” instead of “The data revealed”), to summarize the findings (“We have demonstrated that…”), etc.

As stated by Kedia G, 2023 *“Scientific manuals share a clear consensus about first-person pronouns: “Use them when appropriate”* “, and “*many leading journals encourage the use of first-person pronouns*.”

Although we acknoweldge the remark, we preferred to keep the use of personal pronouns in the manuscript, for better clarity and readability of the overall text.

**2/ Introduction - Include in the introduction section the current state of malaria in Côte d'Ivoire (incidence and mortality).**

Thank you for this relevant comment. We have included the current state of malaria in Côte d’Ivoire in the introduction (l. 99).

**3/ Results - I understand that the focus of this study is on malaria vectors. However, i suppose that, you collected extremely many Culex and Aedes mosquitoes. Their potential role in disease transmission, habitat competition is very important. I suggest that this be taken into account in the results.**

Thanks for your remark. As pointed out, this study focuses on malaria and their vectors, *Anopheles* mosquitoes. Nevertheless, some statistics on the overall diversity and abundance of mosquitoes species collected in the frame of the REACT project (whose entomological data are used in this study) can be found in Zogo et al., 2019a (for the first 5 entomological surveys). As it can be noted, although some *Aedes*, *Culex*, *Mansonia* and *Uranotaenia* mosquitoes were collected, the vast majority of mosquitoes collected actually belonged to the Anopheles genus (94% of the total). The abundance and overall impact of the other mosquito species can therefore be considered negligeable compared to malaria vectors.

To clarify this, we added a sentence in the revised manuscript, stating that more than 90% of the total mosquitoes collected were Anopheles (l. 292).

**4/ Figures 2, 4, 5 and 6: Figures legend and axis are not clear.**

Thank you for pointing out that the figures were not all clear. We have revised the figures so that the legend and axis are more clear and readable.

**References**

Kedia, G. (2023, December 19). *First-person pronouns in scientific articles*. A Brilliant Mind. <https://abrilliantmind.blog/first-person-pronouns-in-scientific-articles/> (accessed 2024, August 5)

Soma D D, Zogo B, Taconet P, Mouline K, Ahoua Alou L P, Dabiré R K, Amanan Koffi A, Pennetier C, Moiroux N (2023). Anopheles collections in the health districts of Korhogo (Côte d'Ivoire) and Diébougou (Burkina Faso) (2016-2018). IRD - Institute of Research for Development. Sampling event dataset <https://doi.org/10.15468/v8fvyn> accessed via GBIF.org on 2024-02-14.

Taconet P, Porciani A, Soma DD, Mouline K, Simard F, Koffi AA, et al. Data-driven and interpretable machine-learning modeling to explore the fine-scale environmental determinants of malaria vectors biting rates in rural Burkina Faso. Parasites Vectors. 2021 Jun 29;14(1)

Taconet P, Soma DD, Zogo B, Mouline K, Simard F, Koffi AA, Dabiré RK, Pennetier C, Moiroux N (2023). Anopheles sampling collections in the health districts of Korhogo (Côte d’Ivoire) and Diébougou (Burkina Faso) between 2016 and 2018. Gigabyte. <https://doi.org/10.46471/gigabyte.83>.

Taconet P, Dieudonné Diloma Soma, Barnabas Zogo, Karine Mouline, Frédéric Simard, Alphonsine Amanan Koﬃ, Roch Kounbobr Dabiré, Cédric Pennetier and Nicolas Moiroux (2024)

Physiological and behavioural resistance of malaria vectors in rural West-Africa: a data mining study to address their ﬁne-scale spatiotemporal heterogeneity, drivers, and predictability, Peer

Community Journal, 4: e11. <https://doi.org/10.24072/pcjournal.367>

Wordvice KH (2023, January 30). *Can you use first-person pronouns (I/we) in a research paper?*. Wordvice. <https://blog.wordvice.com/first-person-pronouns-in-research-paper/> (accessed 2024, August 5)

Zogo et al. (2019a). Anopheles bionomics, insecticide resistance mechanisms, and malaria transmission in the Korhogo area, northern Côte d’Ivoire: a pre-intervention study. In Parasite (Vol. 26, p. 40). EDP Sciences. https://doi.org/10.1051/parasite/2019040

Zogo B, Koffi AA, Alou LPA, Fournet F, Dahounto A, Dabiré RK, et al. (2019b) Identification and characterization of Anopheles spp. breeding habitats in the Korhogo area in northern Côte d’Ivoire: a study prior to a Bti-based larviciding intervention. Parasit Vectors. 2019 Mar 27;12(1):146. <https://doi.org/10.1186/s13071-019-3404-0>
